# Calibration approaches for fluorescence lifetime applications using time-domain measurements

**DOI:** 10.1515/mim-2024-0031

**Published:** 2025-03-13

**Authors:** Anca Margineanu

**Affiliations:** Advanced Light Microscopy, 28341 Max-Delbrück-Centrum für Molekulare Medizin in der Helmholtz-Gemeinschaft, Robert-Rössle-Straße 10, 13125 Berlin, Germany

**Keywords:** FLIM, instrument response function (IRF), reference dyes

## Abstract

This tutorial focuses on presenting experimental protocols for acquiring instrument response functions (IRF) and for calibrating the instruments using reference dyes with validated lifetime in time-domain fluorescence lifetime measurements. Step-by-step preparation of different samples used for the calibrations (scatter solutions, crystals generating second harmonic signal and reference dyes) and the corresponding instrument settings in one- and two-photon excitation are explained. The expected shape of the IRF curves and reference decays are shown using experimentally acquired examples, followed by troubleshooting of the instruments when the expected results are distorted. The discussions focus on the importance of using IRF and reference dyes for adjusting the acquisition parameters of the time-resolved instrument, for data analysis and for comparison and extrapolation of lifetime values between different biological systems.

## 1 Introduction

**Figure 1:**
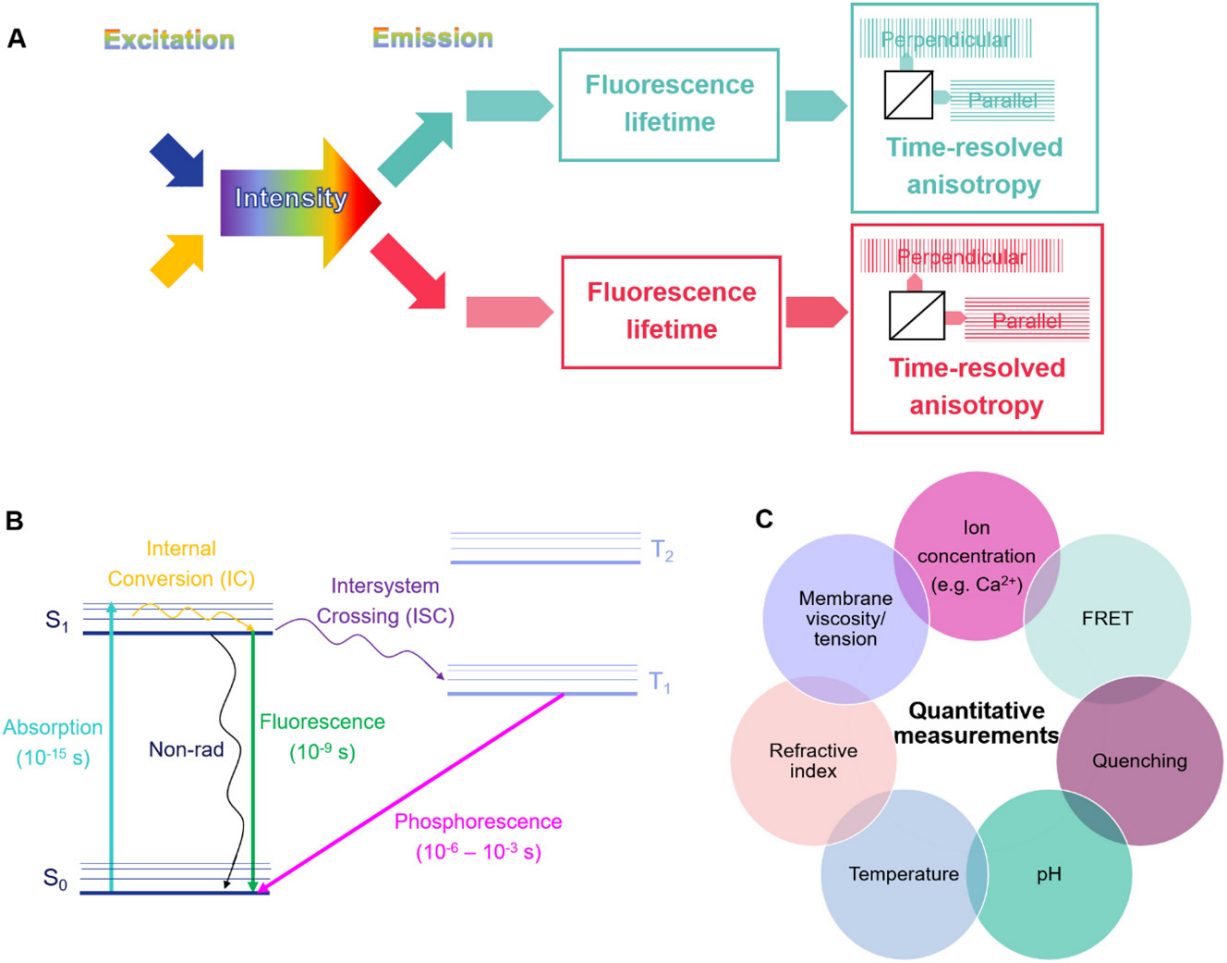
Overview of fluorescence lifetime. (A) Diagram of a multiparameter fluorescence experiment (see text for details). (B) The Jablonski diagram shows the different pathways that can be followed by a fluorescent molecule when returning from the excited to the ground state: internal conversion between vibrational levels within the excited state (IC), non-radiative decay (Non-rad), fluorescence, intersystem crossing between the singlet and the triplet excited state (ISC), phosphorescence. (C) Examples of physical and chemical parameters that can be quantitated using fluorescence lifetime.

Fluorescence techniques have developed significantly in the last decades and are used nowadays for numerous applications in biology, as well as other domains such as medicine, material science or chemistry [1], [2], [3], [4]. Most of the time, measurements rely on the detection of fluorescence intensity, but other fluorescence parameters can be simultaneously obtained in a multiparameter experiment (Figure 1A). Beside intensity, the fluorescence lifetime of a specific fluorophore can be determined, giving information on the local environment of the fluorophore and on molecular interactions (see below). The emitted photons can be further split with respect to the polarisation directions (parallel and perpendicular) to analyse the time-resolved anisotropy decay, which gives information on the rotational time of the fluorophore, as well as on specific interactions such as binding [5], clustering [6], hetero- and homo- FRET (Förster resonant energy transfer) [7], [8]. Another dimension that can be added to multiparameter experiments is the spectral channel, allowing combination of fluorophores with different excitation and emission wavelengths to be detected simultaneously or sequentially in the same sample. In the additional spectral channels, the fluorescence lifetime and the anisotropy decay can be further measured. However, analysing confidently lifetimes and anisotropy decays after splitting the signal in a microscope experiment in multiple channels depends on the photon budget per pixel and is especially challenging when acquiring images at fast rate to follow live cellular processes.

The fluorescence lifetime is a measure of the duration of the excited state, which is a function of the rate constant values that are driving the molecule back from the excited state to the ground state (equation (1) and Figure 1B):

(E1) $$\tau =\frac{1}{k_{f}+k_{nr}+k_{\mathrm{ISC}}}$$

where *τ* = fluorescence lifetime, *k*_*f*_ = fluorescence rate constant, *k*_*nr*_ = non-radiative constant, *k*_*ISC*_ = intersystem crossing rate constant driving the molecule to the triple state.

These constants do not depend only on the internal structure of the fluorescent molecule, but also on the external conditions. For example, *k*_*f*_ and *k*_*nr*_ are strongly dependent on the polarity of the solvent/medium, while *k*_*ISC*_ can be influenced by the presence of molecular oxygen, which has a triplet ground state. Thus, the fluorescence lifetime has a strong dependence on the immediate environment of the fluorescent molecule and can be used to detect quantitatively either physical parameters of the molecular environment (e.g. temperature, refractive index, viscosity, lipid membrane tension etc.) or the presence of chemical factors (e.g. pH, ions, proximity of other molecules via FRET or quenching) (Figure 1C).

On the other hand, in a given environmental condition, the lifetime of a fluorophore in the excited state has a unique value, which should be independent on the instrument or on the technique used for detection, on the excitation intensity and – within limits – on the fluorophore concentration. This characteristic is an advantage for quantitative measurements in thick tissues, because quantitating the fluorescence intensity can be heavily influenced by the light scattering and absorption in these conditions. At the same time, the specific value of the fluorescence lifetime in a given condition allows quantitative comparison between systems ranging from nano-to micro- and mesoscale. Thus, results obtained in single molecule and solution measurements could be extrapolated to cells, tissues or whole organism.

To make such comparisons meaningful, it is necessary to ensure – on the one hand – that the environmental conditions of a fluorescent molecule are as close as possible in different systems. For example, when comparing FLIM-FRET results, the same FRET biosensors should be used for single molecule, solution and cell measurements at the physiologic cellular pH.

On the other hand, it is necessary to carefully calibrate the instruments that are used for different measurements to make sure that the same lifetime value is detected in a given condition, independent of the instrument (e.g. fluorescence lifetime spectrometer or microscope).

This tutorial will focus on performing calibrations for time-domain instruments, based on time correlated single photon counting (TCSPC) or time gating techniques.

**Figure 2:**
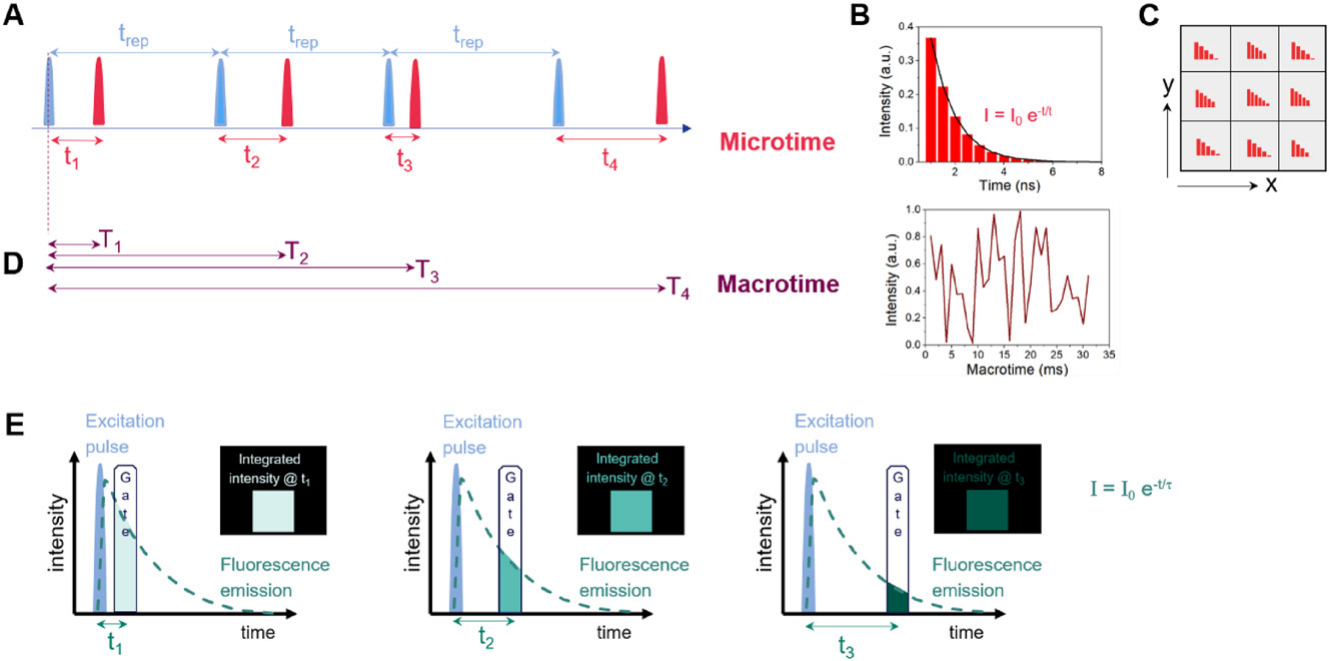
Diagrams of time-resolved fluorescence lifetime instruments. (A–D) Principle of the time correlated single photon counting (TCSPC). (A) The arrival time of the fluorescence photons (red) is measured relative to the laser pulse (blue) and is referred as the microtime. (B) Example of a histogram of photon arrival times fitted with an exponential function to find the fluorescence lifetime. (C) Histograms built in every pixel of a microscope image are used to reconstruct a fluorescence lifetime image (FLIM). (D) Binning the arrival time of photons relative to the start of the experiment are used to build fluorescence intensity traces. (E) Principle of the time-gated technique. An electric input is applied for a short time (ps-ns) on a gated optical intensifier (GOI), allowing an image to be acquired on its phosphorus screen, which is then transferred to a CCD camera. The electronic gate slides at different delay times relative to the laser pulse, generating images with different intensity. The integrated intensity of each pixel in the series of CCD images is fitted with exponential model functions to find the fluorescence lifetime.

The TCSPC technique uses excitation pulses delivered by a laser with a specific repetition rate. When the first emitted photon from a fluorescent molecule in the system reaches the detector, its arrival time relative to the laser pulse is measured (Figure 2A). This time is in the order of ns (i.e. the average time necessary for the fluorescent molecule to return to the ground state) and it is known as the microtime. The electronic clock is reset and the arrival time of the next fluorescent photon relative to the next laser pulse is again measured and so on. A histogram is then built with all the arrival times acquired within the measurement time (Figure 2B) using different number of bins or channels (typically 256, 1024, 4096). The histogram is then fit with exponential decay models to retrieve the fluorescence lifetime. Such histograms can be acquired from every pixel of a microscope image, generating a fluorescence lifetime image (FLIM) (Figure 2C). If the arrival time of the photons is also measured from the start of the experiment, an intensity trace can be built by binning the photons on the macrotime (Figure 2D).

The TCSPC technique is applied in spectrofluorimeters, as well as laser scanning confocal or multiphoton microscope systems using scanning laser beams and single pixel detectors such as photomultiplier tubes (PMTs), hybrid detectors or avalanche photodiodes (APDs). To measure fluorescence lifetimes in wide field configuration microscopes, TCSPC techniques (e.g. using SPAD arrays) have been developed [9], as well as camera-based time-gated techniques [10], speeding up the acquisition of FLIM images. In the case of time-gated techniques, a device called gated optical intensifier (GOI) is synchronised with the laser pulses and acquires images on a phosphorus screen at selected delay times (in the ps-ns range) relative to the laser pulses. Every image is then transferred to a CCD camera. The fluorescence decay is reconstructed from the intensities of each pixel on the series of images that have been acquired with different delays (Figure 2E) and can be fit with exponential models to retrieve fluorescence lifetimes.

For the time domain techniques, the calibrations entail acquiring the instrument response function (IRF) and measuring the fluorescence lifetime of reference dyes. It is recommended that these measurements (or at least one of them) are performed every day before starting the actual experiment and also at the end of the measurement day, if there is a suspicion that conditions might have changed.

### 1.1 Measuring the instrument response function (IRF)

The time domain techniques rely on the excitation of the fluorescence within the sample with a very short pulse of light. Ideally, this pulse is infinitely short and takes the form of the Dirac delta function in a single time bin [11], [12]. In reality, the photons within the laser pulses come with a characteristic time spread. Additional time delays occur due to the temporal dispersion of photons in optical components, due to the transit time of the electrons within the detector and due to the electronic jitter [11], [12]. This means that the fluorescence photons are not counted by the detection system instantaneously after the emission.

The acquired shape of the experimental fluorescence decay curve of the sample is therefore a convolution between the exponential decay of the fluorophore emission and the instrument response. If the fluorophore has a decay time much longer than the instrument response and the decay has a monoexponential form, the convolution can be done using the theoretical delta function [11]. When the experimental decay is multiexponential and has also short components, the instrument response should be measured [11], to consider all the delays generated by the excitation and the detection systems. This is most often the case of biological experiments due to the complexity of the molecular environments (see Discussions for further details).

The experimental width of the IRF varies from system to system, depending on its components and is typically in the order of tens to hundreds ps. The laser pulses used for excitation have typical widths of hundreds of fs (two-photon lasers) up to tens or hundreds of ps (visible solid-state lasers), while fast electronic jitters can be around 1 ps [12]. The detector transit time is in the order of hundreds of ps for photomultiplier tubes and tens of ps for hybrid detectors, single photon avalanche diodes and the more recently developed silicon photomultipliers [12]. For GOIs, typical gate widths are less than 100 ps [13].

For practical reasons, acquiring the IRF before starting the actual measurement allows to check if the ns time window for acquiring the fluorescence decay is correctly chosen, if the start of the fluorescence decay is appropriately set and if the shape of the IRF corresponds to literature data.

The sample measured for the IRF acquisition should ideally generate photons instantaneously after the laser excitation. Very fast – almost instantaneous – signals can be generated by light reflection (using mirrors) or light scattering by small particles. A drawback discussed in relation to these approaches to generate IRFs is that the photons are detected at the same wavelength as the excitation, while the fluorescence coming from the sample is emitted at longer wavelengths (see Discussions for details).

In multiphoton microscopy, the second harmonic generation (SHG) signal of noncentrosymmetric crystals can be conveniently used to measure the IRF. In this case, the signal is detected at half of the excitation wavelength (typically above 700 nm) and is also not in the range of the fluorescence coming from the sample.

Alternatively, fluorescent dyes with very short decay times can be used to acquire the instrument IRF. The dye can be selected such that the excitation and the emission wavelengths are the same as the ones used for the fluorophore labelling the sample to be measured. However, the list of reference dyes with very short fluorescence lifetimes (in the order of tens of ps) that can be chosen for measuring the IRF is limited. Examples of such dyes that can be used both with one- and two-photon excitation are shown in Table 1.

**Table 1:** List of short lifetime dyes that can be used for IRF measurements using visible and two-photon excitation.

Dye	Excitation wavelength (nm)	Emission wavelength (nm)	Solvent	Lifetime (ps)	Reference
Auramine	400–430	470–530	Methanol	<20	[14]
DASPI^a^	400–460	580–630	Water	11	[15]
Erythrosine B	488–530	550–600	Water	80–90	[16], [17]
Rose Bengal	530–560	570–620	Water	77	[18]
Py1^b^	810	660	Water	36	[19]
LDS798^c^	810	750	Water	27	[19]

^a^trans-4-[4-(dimethylamino)-styryl]-1-methylpyridiniumiodide (DASPI). ^b^1-ethyl-4- (4-(p-dimethylaminophenyl)-1,3-butadienyl)-pyridinium perchlorate. ^c^1-ethyl-2-(4-(pdimethylaminophenyl)-1,3-butadienyl)-quinolinium perchlorate.

To acquire IRFs, the fluorescence lifetime of the reference dyes can be significantly shortened by quenching using concentrated solutions containing heavy atoms such as iodine (I^−^). According to Szabelski et al. [20], the Erythrosine lifetime gets as short as 24 ps in a 5.02 M potassium iodide (KI) solution, while the Rose Bengal lifetime in a 5.66 M KI solution was quenched to 16 ps [18]. This approach is applicable also for dyes with longer lifetimes (in the order of few ns). For example, Fluorescein lifetime was quenched to 17 ps in a solution of NaI 12.2 M and was used for IRF measurement to determine the lifetime of circularly permuted green and yellow fluorescent proteins [21].

(1) Protocol for measuring the IRF using light scattering
  – Use a stock colloidal solution of Ludox (e.g. # 420794 from Sigma-Aldrich/Merck) diluted to 10 % in double distilled water.
  – Put a drop on a glass coverslip, or fill a well of a chamber slide (e.g. # 80806 ibidi multiwell chambers for microscopy), or fill a cuvette (e.g. Hellma # Z805025 from Sigma-Aldrich/Merck) and focus the laser beam into the solution, few micrometres away from the glass surface.
  – Excite and collect the emission at the same wavelength.
    *Note 1:* ***Be very careful with the detectors when measuring scattered light!*** *The intensity of the scattered light is very high and can damage the detectors. Start with extremely low excitation power and increase as necessary*.
    *Note 2: If the microscope settings do not allow overlapping the excitation and emission wavelengths, using a solution of a dye with very short fluorescence lifetime (Protocol (3) below) or a dye solution with a quenched lifetime (Protocol (4) below) is recommended*.
    *Note 3: For FLIM microscopes, the reflected light from a mirror placed at the focal plane of the objective can be used instead of the scattered light from a colloidal solution. Be extremely careful and* ***follow strictly the laser safety rules*** *to avoid any reflection directly into your eyes!*
(2) Protocol for measuring the IRF using the second harmonic generation in multiphoton microscopy
  – Prepare a very concentrated solution of urea (e.g. # U5128 from Sigma-Aldrich/Merck) in a small amount of water by adding and stirring the substance until it no longer dissolves. Place 1–2 drops on a glass slide and let it dry. Seal the crystals with a coverglass on top, using a very thin spacer (e.g. # S24735 from ThermoFisher Scientific). The preparation can be kept for very long time.
  – Focus with the objective on the crystals.
  – Excite the crystals with the two-photon wavelength you use for your experiment and collect the second harmonic signal (SHG) at half of this wavelength.
    *Note 1:* ***Be very careful with the detectors when measuring SHG!*** *The SHG intensity is very high and can damage the detectors. Start with extremely low excitation power and increase as necessary. You can also defocus slightly the image*.
    *Note 2: Collect the SHG signal as narrow as possible around half of the two-photon excitation wavelength, otherwise the IRF can get broader due to the fluorescence emission of possible impurities*.
(3) Protocol for measuring the IRF using the fluorescence emission of a reference dye with a short lifetime
  – Prepare a stock solution of Erythrosine B in ethanol of 2 mM. This can be stored very well sealed for many months at room temperature, preferably in a glassware.
  – Freshly dilute the stock solution in double distilled water to a final concentration 2–10 µM.
    *Note 1: Use very pure Erythrosine B (>95 %, e.g. # 200964 from Sigma Aldrich), spectroscopic grade ethanol (e.g. # 493511 from Sigma Aldrich) and double distilled water*.
    *Note 2: Storing dyes in water for long time can lead to aggregation and lifetime changes. Therefore, it is better to dilute the stock solution just before measurement*.
    *Note 3: Do not prepare the solution with more than 10 µM concentration, as reabsorption processes of the emitted photons can take place, changing the measured lifetime* [22].
  – Put a drop on a glass coverslip or fill a well of a chamber slide (e.g. # 80806 ibidi multiwell chambers for microscopy) or fill a spectroscope cuvette (e.g. Hellma # Z805025 from Sigma-Aldrich/Merck). Focus the laser beam into the solution, few micrometres away from the glass surface.
    *Note 4: Make sure you don’t deep water-immersion objectives into the dye solution when using an upright microscope. The objectives will get contaminated, it will be difficult to clean them and this could affect the measured lifetimes of other probes*.
  – Excite the erythrosine solution at 488 or 514 nm and detect between 550 and 600 nm.
  – The expected lifetime of Erythrosine B in water is 80–90 ps.
(4) Protocol for measuring the IRF by quenching the fluorescence emission of a reference dye using heavy atoms
  – Prepare a 2 mM stock solution of Erythrosine B in ethanol.
  – Freshly dilute the stock solution of Erythrosine B to 10 µM in a 5.02 M solution of KI in double distilled water (see also Szabelski et al. [20]).
*Note 1: Use very pure chemicals: Erythrosine B (>95 %, e.g. # 200964 from Sigma Aldrich), KI (>99 %, e.g. # 196730025 from ThermoFisher Scientific), spectroscopic grade ethanol (e.g. # 493511 from Sigma Aldrich) and double distilled water.*
    *Note 2: Storing dyes in water for long time can lead to aggregation and lifetime changes. Therefore, it is better to dilute the stock solution just before measurement*.
  – Put a drop on a glass coverslip or fill a well of a chamber slide (e.g. # 80806 ibidi multiwell chambers for microscopy) or fill a spectroscope cuvette (e.g. Hellma # Z805025 from Sigma-Aldrich/Merck). Focus the laser beam into the solution, few micrometres away from the glass surface.
    *Note 5: Make sure you don’t deep water-immersion objectives into the dye solution when using an upright microscope. The objectives will get contaminated, it will be difficult to clean them and this could affect the measured lifetimes of other probes*.
  – Erythrosine B can be excited at 488 nm and detected between 550 and 600 nm.
  – The expected quenched lifetime of Erythrosine B is 24 ps.

#### 1.1.1 Expected results

**Figure 3:**
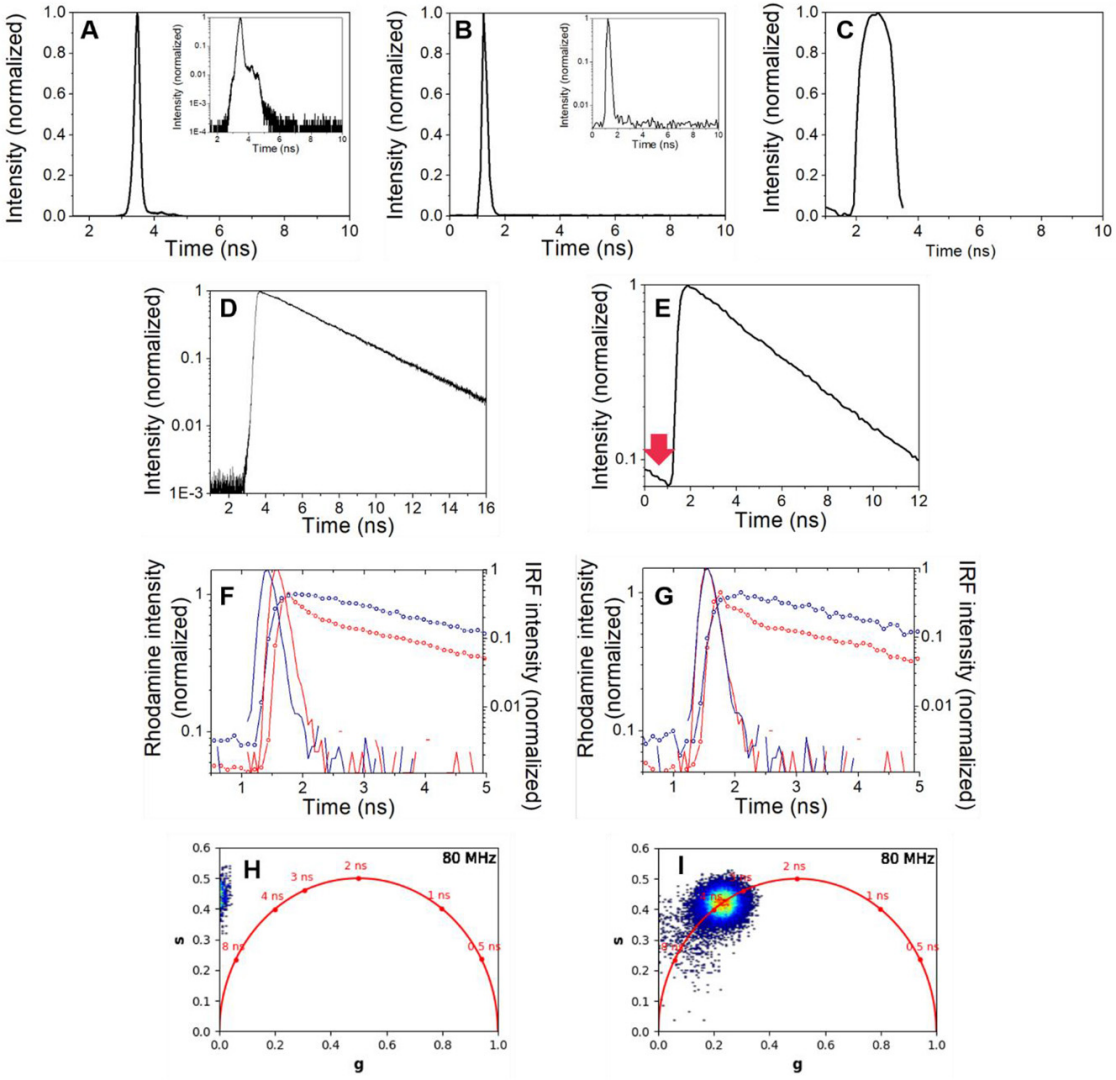
Expected results for measuring the instrument response function (IRF) and standard dyes decay on time-domain fluorescence lifetime systems. (A) An IRF measured using a scatter solution excited at 488 nm on the TCSPC fluorescence spectrometer. (B) An IRF measured using the second harmonic generation of urea crystals on a two-photon microscope. (C) An IRF measured using a non-quenched Erythrosine B solution on a time-gated microscope. (D) Example of a monoexponential decay of Rhodamine 6G in water using a 20 ns time window to allow the complete fluorescence decay. Note the logarithmic intensity scale. (E) Example of a monoexponential decay of Rhodamine 6G in water reconstructed from the FLIM image on a two-photon microscope equipped with a laser with 80 MHz repetition rate, allowing 12.5 ns time window. Note the presence of the last part of the Rhodamine decay in the background before the rising edge, marked by the red arrow (incomplete decay). (F-G) Example of polarisation traces for Rhodamine 6G in water (two-photon microscopy). Note the shift between the parallel (red) and perpendicular (blue) IRF channels in F. The FLIMfit software [23] corrects this difference and shifts the perpendicular decay curve (also in blue) of Rhodamine to analyse correctly the anisotropy decay (G). (H–I) Example of Rhodamine 6G lifetime values within the pixels of the two-photon FLIM image visualised on a phasor plot using the FLUTE software [24]. The calculated lifetime values distribute outside the universal circle (red) without loading the calibration file of the IRF (H), but they appear concentrated around 4 ns on the universal circle after loading the IRF calibration (I), as expected for a monoexponential decay. See Materials and Methods for description of the experimental conditions.

The IRF shape will differ from system to system. In TCSPC based instruments, this shows a prominent peak followed by a small shoulder (Figure 3A and B) with a full width at half maximum (FWHM) between 28 ps and 200–300 ps, depending on the lasers, detectors and the electronic system. In time gated systems, the IRF takes a particular broader shape, mimicking the electronic time gate (Figure 3C).

### 1.2 Measuring the fluorescence lifetime of reference dyes

Beside the IRF, a useful calibration of the fluorescence lifetime systems is to measure a dye with a known value of the decay time. These dyes are selected to have a single exponential decay in given conditions, allowing to check – on the one hand – that the value is reproducible with the user’s instrument and – on the other hand – that no additional exponential components are necessary to fit the decay, introducing artefacts in the subsequent analysis of the experimental data. A list with various reference dyes that can be used across different spectral ranges is available in ref. Boens et al. [16].

Protocol for measuring the lifetime using Rhodamine 6G as a reference dye.

– Prepare a stock solution of Rhodamine 6G in ethanol of 2 mM. This can be stored for many months at room temperature.
  *Obs.: Use very pure Rhodamine 6G (>99 %, e.g. # 252433 from Merck/Sigma Aldrich) and spectroscopic grade ethanol (e.g. # 493511 from Merck/Sigma* *Aldrich)*.
– Freshly dilute 1000x the stock solution in double distilled water to a final concentration of 2 µM.
*Note 1: Storing dyes in water for long time can lead to aggregation and lifetime changes. Therefore, it is better to dilute the stock solution just before measurement.*
  *Note 2: Do not prepare the solution with a concentration higher than 10 µM, as reabsorption processes of the emitted photons can take place, changing the measured lifetime* [22].
– Put a drop on a glass coverslip, or fill a well of a chamber slide (e.g. (e.g. # 80806 ibidi multiwell chambers for microscopy) or fill a cuvette (e.g. Hellma # Z805025 from Sigma-Aldrich/Merck) and focus the laser beam into the solution, few micrometres away from the glass surface.
  *Note 3: Make sure you don’t deep water-immersion objectives into the Rhodamine solution – they will get contaminated and this could affect the measured lifetimes of other probes. Rhodamine can be much brighter than other dyes used in biology and difficult to clean*.
– Excite the solution at 488 nm and detect 500–600 nm. For two-photon experiments, the maximum of excitation is situated around 800 nm, but other wavelengths (±50 nm) can be also used, as the two-photon excitation spectra are generally broad.
– The expected lifetime of Rhodamine 6G is 3.9–4.0 ns.

#### 1.2.1 Expected results

Figure 3D shows an example of a decay measured using Rhodamine 6G in water. To visualize the monoexponential decay, a logarithmic scale was used on the *y* axis. In this way, the graph shows a sharp rising edge and a linear falling edge.

### 1.3 Troubleshooting

Possible problems one may encounter when acquiring IRFs or reference decays are related to:

– the sample preparation (i.e. the concentration of reference dyes is either too low or too high, solutions are not freshly prepared);
– the settings of the microscope light path (i.e. selection of the laser wavelength and power adjustment, selection of dichroic mirrors, emission filters, detectors, shutters);
– the settings of the FLIM acquisition (i.e. setting the correct triggering and delays between the laser and the detector, using the same settings for the IRF and the sample when fitting).

A list of issues that can arise and the corresponding instrument settings to be checked are presented in Table 2.

**Table 2:** Troubleshooting problems when calibrating time domain fluorescence lifetime systems.

Issue	Checklist
No fluorescence signal	Check that the laser shutter is opened Check that the correct combination of laser, dichroic and emission filter is selected Check that the light path is directed to the active detector Check that the correct detector is active Check that the objective is focused within the solution (a few µm above the glass surface) or on the SHG crystals
Too high signal when measuring scatter IRF or SHG	Always start with very low laser power Reduce the laser power if the image is oversaturated Defocus slightly the image of the crystals generating the SHG
Too low signal when using a reference dye	Check that the objective is focused within the solution (a few µm above the glass surface) Increase the laser power Increase the concentration of the dye (but no more than 10 µM) Increase the acquisition time or the exposure time of the camera
No fluorescence decay is observed	Check that the objective is focused within the solution (a few µm above the glass surface) or on the SHG crystals Check that the correct combination of laser, dichroic and emission filter is selected Check that the acquisition trigger is coming from the correct laser (when multiple lasers are in the system) Increase the laser power
The peak of the IRF or reference decay is not visible within the time window of the measurement	Adjust the delay between the laser pulse and the start of the acquisition at the detector, until the rising edge and the peak of the decay become visible, leaving the first channels in the time window for background
The IRF or the reference decay show additional peaks	Clean the objective Check that the objective is focused within the solution (a few µm above the glass surface) or on the SHG crystals Check the characteristics of the dichroic and the emission filter to make sure that they do not allow the excitation light to pass Change the combination of the dichroic and the emission filter to avoid possible back reflections of light Check that there is no reflective surface under the sample (all metal holder parts should be black) Check the connection of the detectors, as specified by the manufacturer, to avoid electronic cross-talk Contact the manufacturer if the problem persists
The reference cannot be fit correctly with a monoexponential decay or is not found on the universal circle (phasor plot)	Prepare a fresh solution in double distilled water by diluting the stock in ethanol Do not use concentrations above 10 µM Check the characteristics of the dichroic and the emission filter to make sure that they do not allow the excitation light to pass Make sure you use the IRF acquired with the same settings for fitting

## 2 Discussions

To accurately determine fluorescence decay times and also to make comparisons and extrapolations between samples measured with different instruments, reference samples are essential. Recording an IRF is necessary when analysing FLIM data using model fitting algorithms based on the nonlinear least-squares iterative reconvolution method (minimisation of χ^2^ or maximum likelihood estimator, MLE). When applying algorithms that do not require a fitting model such as the phasor plot (see below), either IRF or a monoexponential reference can be equally used ([24]). Other methods that deconvolve the time-resolved data without any IRF assumption (e.g. based on Laguerre expansion [25]) can benefit from the calibration of the instrument with reference dyes to validate the results.

Measuring the IRF and reference dyes at the beginning of the experiment also helps to set the initial parameters for acquisition. The time window for acquiring a fluorescence decay should be 5–6 longer than the expected lifetime of the sample to allow the complete decay of the excited molecules (Figure 3D). However, the time window that can be set in an experiment depends on the pulse repetition rate of the laser. For example, a pulsed diode laser with a repetition rate of 40 MHz will offer a time window of 25 ns between two consecutive pulses, which is enough for fluorophores with lifetimes around 4 ns. This can be adjusted if the expected lifetimes are around 2 ns to avoid the utilisation of too many channels containing only background photons. For multiphoton measurements, the two-photon fs-lasers have a typical repetition rate of 80 MHz, and therefore the time window is only 12.5 ns. In this case, the decay of a fluorophore with a lifetime of 4 ns is not completed before the next pulse arrival (Figure 3E), which can introduce errors in the determination. The time window can be extended by using a pulse picker instrument, to the detriment of a reduced number of photons collected per time unit. However, recent software for FLIM data analysis use algorithms for compensating incomplete decays of fluorophores [23], [26].

The starting point of the decay can be set by adjusting the time delay between the laser pulse and the detector and it should not be placed in the first channels of the time window. In these channels, the background and the full rising edge of the decay should be visualised, to correctly fit the experimental decay by convolution with the IRF.

Reconvolution with an experimental IRF when analysing multiexponential decays helps to define precisely the start of the decay and to estimate short decay times with better precision than using only a tail-fit of the decaying part of the experimental curve [27]. Multiexponential decays are most often encountered in biological experiments due to the complexity of the molecular environments, even when labelling was performed with dyes considered to have monoexponential lifetimes in certain conditions. Additionally, fluorophores such as fluorescent proteins can have intrinsic multiexponential behaviour.

Fitting fluorescence intensity decays with experimental IRFs also makes the comparison and extrapolation of results between different experiments and instruments more reliable. For example, the average lifetime of the donor in an intramolecular FRET Ca^2+^ biosensor was calibrated for different Ca^2+^ concentrations in cytosolic extracts using a fluorimeter [28] and further used to determine the intracellular Ca^2+^ concentration upon cellular activation in a FLIM microscope [8]. In this case, the extrapolation of results was possible because the same biosensor was used to transfect the cells in both cases, the pH of the cytosolic preparation and of the cytoplasm of live cells had the same value and IRFs were measured to analyse the decays in both instruments.

Defining precisely the start of the decay is an important issue also for time-resolved polarisation decays, as small shifts can exist between the parallel and the perpendicular detection channels (Figure 3F). This should be corrected in order to fit correctly anisotropy decays, especially for short rotational correlation times (Figure 3G).

Most of the times, the analysis software requires that the IRF and the experimental decays are measured with the same time window and number of channels, but other software (e.g. FLIMfit [23]) allow fitting using a higher number of channels for the IRF. This can help to detect with improved precision the start of the decay.

It is important to acquire the IRF also because its width – together with the bin width and the number of acquired photons – determines the shortest resolvable decay time and the smallest change in the decay time that can be detected [29].

As already mentioned, using the same spectral bands for signal detection of both the IRF and the sample is considered a better option than using scattered light because the temporal response of the photodetectors shows dependence on the spectral regions due to different energies of the incoming photons generating electrons [30], [31]. Because only a limited range of dyes with very short lifetime are available, it was proposed to measure instead a reference dye (e.g. Rhodamine 6G) and either do reconvolution considering its long lifetime, or derive an IRF based on the rising edge of its monoexponential decay [23]. For two-photon microscopy IRFs, it is possible to utilise gold nanoparticles with extended spectral emission based on plasmon resonance to replace the typical SHG signal used for IRF acquisition [31].

A problem when measuring the experimental IRF is the background accumulation. Therefore, IRF background subtraction is necessary to be implemented in the analysis software [23], [32]. Also, the lifetime of the dye (e.g. Erythrosine B) should be considered in the fitting model for the convolution with the experimental decay [23].

Phasor plot analysis is recently an alternative method to analyse fluorescence decays acquired in time domain measurements. Although it is considered an easier approach than the exponential fit because no model must be assumed, recording IRF or reference dyes is still essential. If only the arrival photon times are considered, the data cloud may not be found on the universal circle (for monoexponential decays) or inside the circle (for multiexponential decays) and the lifetimes will not be correctly estimated (Figure 3H and I, [24]).

## 3 Conclusions

Calibrating the time domain FLIM instruments using IRF or reference dyes is a vital part of an experimental workflow for quantitative measurements. This calibration should help essentially to precisely identify the start of the decay, which is especially important for analysing the fluorescence decay with multiple components encountered in biological systems, as well as for time-resolved anisotropy analysis. It also makes the comparison and extrapolation of results between different experiments more reliable.

Measuring either a scattering solution or a solution containing a short decay time reference dye, as well as using the SHG in two-photon experiments, are all valid approaches to record the IRF and have been widely applied in literature. When using software that estimates the IRF based on the rising edge of an experimental fluorescence decay profile, it is important to check if the fitting gives the same values when using a measured IRF. The results can be improved if the IRF estimation is based on a monoexponential decay of a reference dye with long lifetime and the same IRF curve is used for fitting all the data within an experiment.

Using reference dyes helps confirming that the instrument measures the specific value of their lifetime and that no additional components are observed, e.g. due to leaking scattered excitation light, additional reflections from filters or dichroics or electronic cross-talk in multiple detection channels.

## 4 Materials and methods

The scatter IRF and the Rhodamine 6G decay in Figure 3A–D were acquired as calibration data for experiments performed using a time-resolved spectrometer for the publication [33] (calibration data not shown in this publication). For a full description of the TCSPC spectrometer, see Manning et al. [34]. The IRF was measured using a scattering solution of Ludox (420794 from Sigma-Aldrich) filled in a quartz cuvette for spectroscopy (Hellma), excited at 488 nm. Rhodamine 6G (252433 Sigma-Aldrich) was diluted to 2 µM in double distilled water from a 2 mM stock solution in ethanol spectroscopic grade and was used to fill a quartz cuvette for spectroscopy (Hellma). Excitation was done at 488 nm and collection at 560 nm.

The IRF measured using non-quenched Erythrosine B in Figure 3C was acquired as calibration data for the publication [35] (data not shown in this publication) using a time-gated FLIM microscope. Excitation was done at 472/30 nm and detection at 520/35 nm. For a full description of this setup, see Talbot et al. [10]. 200 µl of a 10 µM Erythrosine B solution (200964 from Sigma Aldrich) in double distilled water were added to a well in a 96-multiwell plate (655076 from Greiner Bio-One).

All other data in Figure 3 were acquired using a two-photon microscope (Trimscope II, LaVision Biotec/Miltenyi) and the TCSPC technique. A pulsed Ti:Sapph laser (Chameleon, Coherent, USA) was tuned at 780 nm. A half-wave plate and a polariser are used to control the laser power. Imaging was done using a 10x/NA 0.6 water dipping Olympus objective. Detection was collected at 520/40 nm for Rhodamine 6G or at 385/26 nm for IRF using a hybrid detector. The IRF was acquired by measuring the second harmonic signal (SHG) from urea crystals as described in Protocol (2). The freshly diluted Rhodamine G solution in water (2 µM) was filled in a well of an 8-well slide (80806 from ibidi), which was covered with a coverglass (no. 1.5, CLS2980245 from Sigma-Algrich) to avoid the objective of the upright microscope to touch the fluorescent solution. For time-resolved polarisation measurements, the signal was split using a polarising beam cube between two hybrid detectors.
